# People with dyslexia and heart, chest, skin, digestive, musculoskeletal, vision, learning, speech and mental disorders were more dissatisfied with neighbourhoods: Scottish Household Survey, 2007–2008

**DOI:** 10.1007/s11356-016-7585-1

**Published:** 2016-09-14

**Authors:** Ivy Shiue

**Affiliations:** 1 Faculty of Health and Life Sciences, Northumbria University, Newcastle upon Tyne, England NE1 8ST UK; 2 Alzheimer Scotland Dementia Research Centre, University of Edinburgh, Edinburgh, UK

**Keywords:** Dyslexia, Chronic disease, Mental health, Adult health, Built environment, Neighbourhood satisfaction, Cognition performance

## Abstract

Rarely do we know the perception toward neighbourhoods in people specifically with health conditions. Therefore, the aim of the present study was to understand the perception toward neighbourhoods among adults with a series of the existing health conditions in a country-wide and population-based setting. Data were retrieved from and analysed in Scottish Household Survey, 2007–2008. Information on demographics, self-reported health conditions and perception toward neighbourhoods and the surrounding facilities was obtained by household interview. Analysis including chi-square test, *t* test and logistic regression modelling were performed. Of 19,150 Scottish adults (aged 16–80) included in the study cohort, 1079 (7.7 %) people were dissatisfied with their living areas; particularly for those who experienced harassment (15.4 %), did not recycle or with dyslexia, chest, digestive, mental and musculoskeletal problems. Twenty to forty per cent reported common neighbourhood problems including noise, rubbish, disputes, graffiti, harassment and drug misuse. People with heart or digestive problems were more dissatisfied with the existing parks and open space. People with arthritis, chest or hearing problems were more dissatisfied with the waste management condition. People with dyslexia were more dissatisfied with the existing public transportation. People with heart problems were more dissatisfied with the current street cleaning condition. People with hearing, vision, speech, learning problems or dyslexia were also more dissatisfied with sports and recreational facilities. People with heart, chest, skin, digestive, musculoskeletal, vision, learning, speech and mental disorders and dyslexia were more dissatisfied with their current neighbourhood environments. Upgrading neighbourhood planning to tackle social environment injustice and put pleasant life experience as priorty would be suggested.

**Graphical abstract:**
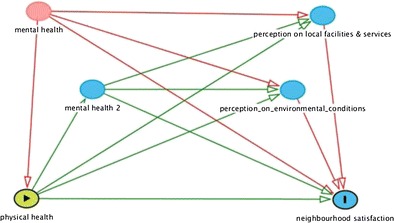
interrelations of individual health and neighbourhood health

interrelations of individual health and neighbourhood health

## Introduction

### Evidence before this study

Effects of neighbourhoods on human health have been described, and the existing literature has documented that problematic neighbourhoods could predict different social and health issues such as drug-use patterns into middle adulthood from local to global levels (Reitzel 2012). Apart from the known risks such as deprivation and crime rates, little is known on the perceived neighbourhood problems which would be of value incorporating place-based approach to optimise health and well-being in regional or national environments (Warr et al. 2009). From a socio-psychological point of view, it is known that life satisfaction falls under the broad area of subjective well-being (Bowling et al. 1993). Similarly, neighbourhood satisfaction as an environmental factor of human living society could refer to an overall assessment of one’s satisfaction toward his/her living surrounding which would be an important indicator of subjective well-being as well and consequently quality of life (Chapman and Beaudet 1983). Recently, it has been observed that perceived noise, water, rubbish, traffic and etc. among adults and the very old across Europe have led to poor mental health (Shiue 2014a). Poor perception toward neighbourhoods has also been found to be associated with emotional and behavioural problems in adolescents in the UK (Shiue 2014b).

### Knowledge gap

Following this context, however, rarely do we know the perception toward neighbourhoods in people specifically with health conditions due to a lack of research evidence in this area. Therefore, the aim of the present study was to understand the perception toward neighbourhoods among adults with a series of the existing health conditions and disabilities in a country-wide and population-based setting.

## Methods

### Study sample

Scottish Household Survey (more details via http://www.scotland.gov.uk/topics/statistics/16002) has been a country-wide, population-based, multi-year (every 2 years) study since 1999. It covers housing, social justice and transport to effectively evaluate the composition, characteristics, attitudes and behaviours of households and individuals at national and sub-national level in Scotland (more details via http://www.scotland.gov.uk/Topics/Statistics/16002/SurveyOverview/). It aims to allow the relationships between social variables within households to be examined, supporting cross-departmental and inter-departmental policies to optimise the Scottish welfare. In the current analysis, the most recent publicly available data, the 2007–2008 cohort (more details via http://www.scotland.gov.uk/Topics/Statistics/16002/DataAccesAgreements), on demographics, self-reported health conditions and perception toward neighbourhoods and the surrounding facilities among Scottish adults was obtained by household interview.

### Variables and analyses

Self-reported health conditions included arthritis, speech impairment, chest or breathing problems; diabetes; difficulty hearing; difficulty seeing (even when wearing glasses/lenses); dyslexia; epilepsy; heart, blood pressure or circulatory problems; learning or behavioural problems; mental health problems; problems or disability related to arms, hands, legs, feet, back or neck; severe disfigurement, skin condition, allergies stomach, liver, kidney or digestive problem; some other progressive disability or illness or some other health problem or disability (Question: Which of the conditions listed on this card best describes the ill-health or disability that the person has?). Study outcome variables included perception toward the way local agency dealing with neighbourhood issues, sports/leisure facilities, library facilities, museum/gallery facilities, theatre facilities, parks and open space, local health services, police service, fire service, refuse collection, local schools, social care or social work services, public transport and street cleaning (Question: Overall, how satisfied or dissatisfied are you with each of these services?). Potential covariates including age, sex and experience of harassment were adjusted. Effects were estimated by using odds ratios (OR) or relative risk ratios (RRR) and 95 % confidence intervals (CI) depending on the study outcome variables being binary or categorical, with *P* < 0.05 considered statistically significant. Statistical software STATA version 13.0 (STATA, College Station, Texas, USA) was used to perform all the analyses.

### Ethics consideration

Since there is only secondary data analyses employed without any participant personal information identified by extracting statistical data from the UK Data Archive website in the present study, no further ethics approval for conducting the present study is required (more details via http://www.ethicsguidebook.ac.uk/Secondary-analysis-106).

## Results

### Descriptive statistics

Of 19,150 Scottish adults (aged 16–80) included in the study cohort, 1079 (7.7 %) people were dissatisfied with their living areas; particularly for those who had experienced harassment (15.4 % of all adults), did not do recycling or with dyslexia, chest, digestive, mental and musculoskeletal problems. 20–40 % reported common neighbourhood problems including noise, rubbish, disputes, graffiti, harassment and drug misuse (see Table 1). Women or people with a younger age could be more dissatisfied with their neighbourhoods, compared with their counterparts. Geographically, the top 3 sub-regions with higher proportion of dissatisfaction with neighbourhoods are Greater Glasgow and Cly (11.2 %), Lanarkshire (9.1 %) and Forth Valley (8.2 %).

**Table 1:** Characteristics of the Scottish adults aged 16–80 (*n* = 19,150) and their neighbourhoods

	A good place to live (*n* = 13,054, 92.4 %)	A poor place to live (*n* = 1079, 7.7 %)	*P* value
Sex			
Male	6615 (93.7 %)	446 (6.3 %)	<0.001
Female	6439 (91.1 %)	633 (9.0 %)	
Age	54.8 ± 17.3	46.1 ± 16.9	<0.001
16–39	1896 (86.9 %)	435 (13.1 %)	
40–79	8941 (93.7 %)	601 (6.3 %)	
80	1217 (96.6 %)	43 (3.4 %)	
Ethnicity			
White	12,818 (92.4 %)	1057 (7.6 %)	0.644
Others	229 (91.6 %)	21 (8.4 %)	
Ever harassment experience			
No	11,263 (94.2 %)	693 (5.8 %)	<0.001
Yes	1780 (82.2 %)	385 (17.8 %)	
How common are neighbourhood problems?			
Noisy neighbours	859 (65.5 %)	453 (34.5 %)	<0.001
No	12,133 (95.1 %)	623 (4.9 %)	
Vandalism, graffiti	1586 (69.4 %)	699 (30.6 %)	<0.001
No	11,389 (96.8 %)	373 (3.2 %)	
Rubbish around	3198 (80.3 %)	785 (19.7 %)	<0.001
No	9806 (97.1 %)	292 (2.9 %)	
Neighbour disputes	433 (57.7 %)	317 (42.3 %)	<0.001
No	12,455 (94.4 %)	736 (5.6 %)	
Intimidating or harassing others	1037 (65.0 %)	559 (35.0 %)	<0.001
No	11,868 (96.1 %)	486 (3.9 %)	
Drug misuse	1204 (65.4 %)	637 (34.6 %)	<0.001
No	10,845 (97.2 %)	318 (6.9 %)	
Rowdy behaviours	1585 (68.4 %)	733 (31.6 %)	<0.001
No	11,346 (97.1 %)	336 (2.9 %)	
Abandoned/burnt vehicles	181 (60.9 %)	116 (39.1 %)	<0.001
No	12,713 (93.1 %)	938 (6.9 %)	
Recycling			
Glass, jars	6791 (94.9 %)	368 (5.1 %)	<0.001
None	2994 (87.8 %)	416 (12.2 %)	
Plastic bottles	568 (94.1 %)	358 (5.9 %)	<0.001
None	4116 (90.6 %)	426 (9.4 %)	
Metal cans	5877 (94.5 %)	341 (5.5 %)	<0.001
None	3907 (89.8 %)	443 (10.2 %)	
Paper, cardboard	7948 (94.2 %)	493 (5.8 %)	<0.001
None	1837 (86.3 %)	291 (13.7 %)	
Local health boards			
Grampian	1329 (95.5 %)	62 (4.5 %)	<0.001
Tayside	947 (92.6 %)	76 (7.4 %)	
Fife	914 (92.4 %)	75 (7.6 %)	
Lothian	1809 (92.3 %)	152 (7.8 %)	
Borders	286 (95.0 %)	15 (5.0 %)	
Forth Valley	763 (91.8 %)	68 (8.2 %)	
Lanarkshire	1066 (90.9 %)	107 (9.1 %)	
Ayrshire and Arran	882 (92.1 %)	76 (7.9 %)	
Dumfries and Galloway	444 (94.7 %)	25 (5.3 %)	
Orkney	266 (98.9 %)	3 (1.1 %)	
Shetland	219 (98.7 %)	3 (1.4 %)	
Western Isles	228 (97.4 %)	6 (2.6 %)	
Greater Glasgow and Cly	2889 (88.8 %)	366 (11.2 %)	
Highland	1012 (95.7 %)	45 (4.3 %)	

### Analytical statistics

In Table 2, associations between existing health conditions and perception toward the way that the local agency dealt with neighbourhood issues are shown while in Tables 3, 4, 5, 6, 7, 8, 9, 10, 11 and 12, associations between existing health conditions and perception toward specific neighbourhood facilities are presented accordingly. In general, people with heart or digestive problems were more dissatisfied with the existing parks and open space. People with arthritis, chest or hearing problems were more dissatisfied with the refuse collection condition. People with dyslexia were more dissatisfied with the existing public transportation. People with heart problems were more dissatisfied with the current street cleaning condition. People with hearing, vision, speech, learning problems or dyslexia were also more dissatisfied with sports and recreational facilities.

**Table 2:** Associations between health conditions and neighbourhood satisfaction in Scottish adults aged 16–80

	A good place to live (*n* = 13,054, 92.4 %)	A poor place to live (*n* = 1079, 7.7 %)	OR-1 (95 % CI)	*P* value	OR-2 (95 % CI)	*P* value
Speech impairment	41 (87.2 %)	6 (12.8 %)	1.26 (0.52–3.04)	0.612	0.95 (0.35–2.61)	0.922
No	3653 (90.0 %)	408 (10.1 %)	1.00		1.00	
Chest/breathing problem	626 (87.4 %)	90 (12.6 %)	1.46 (1.13–1.88)	0.004	1.33 (0.97–1.81)	0.077
No	2068 (90.5 %)	324 9.6 %)	1.00		1.00	
Diabetes	453 (91.2 %)	44 (8.9 %)	1.03 (0.73–1.44)	0.871	1.38 (0.94–2.03)	0.100
No	3241 (89.8 %)	370 (10.3 %)	1.00		1.00	
Difficulty hearing	302 (90.4 %)	32 (9.6 %)	1.33 (0.90–1.97)	0.156	1.16 (0.70–1.90)	0.558
No	3392 (89.9 %)	382 (10.1 %)	1.00		1.00	
Difficulty seeing	280 (90.9 %)	28 (9.1 %)	1.11 (0.73–1.68)	0.625	1.04 (0.63–1.71)	0.886
No	3414 (89.8 %)	386 (10.2 %)	1.00		1.00	
Dyslexia	22 (66.7 %)	11 (33.3 %)	2.27 (1.05–4.90)	0.038	2.63 (1.04–6.62)	0.040
No	3671 (90.1 %)	403 (9.9 %)	1.00		1.00	
Epilepsy	86 (81.1 %)	20 (18.9 %)	1.32 (0.79–2.22)	0.294	1.54 (0.80–2.93)	0.193
No	3608 (90.2 %)	394 (9.9 %)	1.00		1.00	
Heart/circulatory problem	1267 (91.5 %)	118 (8.5 %)	1.18 (0.93–1.51)	0.170	1.24 (0.94–1.65)	0.134
No	2427 (89.1 %)	296 (10.9 %)	1.00		1.00	
Learning/behavioural problem	25 (89.3 %)	3 (10.7 %)	0.48 (0.14–1.63)	0.240	0.28 (0.04–2.21)	0.228
No	3669 (89.9 %)	411 (10.1 %)	1.00		1.00	
Mental health problem	350 (78.1 %)	98 (21.9 %)	1.57 (1.19–2.06)	0.001	1.21 (0.87–1.69)	0.266
No	3344 (91.45)	316 (8.6 %)	1.00		1.00	
Disability: arms, hands	421 (87.3 %)	61 (12.7 %)	1.39 (1.03–1.87)	0.030	1.23 (0.85–1.77)	0.265
No	3273 (90.3 %)	353 (9.7 %)	1.00		1.00	
Disability: legs, feet	1019 (91.1 %)	100 (8.9 %)	1.01 (0.79–1.29)	0.911	1.09 (0.82–1.45)	0.559
No	2675 (89.5 %)	314 (10.5 %)	1.00		1.00	
Disability: neck, back	642 (87.8 %)	89 (12.2 %)	1.29 (1.00–1.66)	0.052	1.35 (1.00–1.82)	0.051
No	3052 (90.4 %)	325 (9.6 %)	1.00		1.00	
Severe disfigurement, skin condition or allergies	74 (84.1 %)	14 (15.9 %)	1.57 (0.86–2.85)	0.139	1.16 (0.55–2.46)	0.690
No	3620 (90.1 %)	400 (10.0 %)	1.00		1.00	
Severe stomach, liver, kidney or digestive problems	298 (85.4 %)	51 (14.6 %)	1.54 (1.11–2.13)	0.009	1.49 (1.02–2.09)	0.041
No	3396 (90.3 %)	363 (9.7 %)	1.00		1.00	
Some other progressive disability or illness	211 (89.8 %)	24 (10.2 %)	1.04 (0.67–1.63)	0.847	1.15 (0.68–1.95)	0.593
No	3483 (89.9 %)	390 (10.1 %)	1.00		1.00	
Some other health problem or disability	493 (89.5 %)	58 (10.5 %)	0.96 (0.71–1.30)	0.808	0.98 (0.69–1.39)	0.916
No	3201 (90.0 %)	356 (10.0 %)	1.00		1.00	
Arthritis	1099 (90.9 %)	110 (9.1 %)	1.22 (0.95–1.56)	0.112	1.16 (0.87–1.56)	0.321
No	2595 (89.5 %)	304 (10.5 %)	1.00		1.00	

*OR-1* adjusted for age and sex, *OR-2* adjusted for age, sex and ever harassment

**Table 3:** Associations between health conditions and perception toward dealt neighbourhood issues in Scottish adults aged 16–80

	Satisfaction (*n* = 6169, 43.6 %)	Neutral (*n* = 5465, 38.6 %)	RRR (95 % CI)	Dissatisfaction (*n* = 2526, 17.8 %)	RRR (95 % CI)
Ever harassment	709 (32.6 %)	713 (32.8 %)	1.10 (0.98–1.23)	754 (34.8 %)	3.03 (2.69–3.41)
No	5458 (45.6 %)	4746 (39.6 %)		1771 (14.8 %)	
Speech impairment	22 (45.8 %)	16 (33.3 %)	0.93 (0.49–1.79)	10 (20.8 %)	0.83 (0.38–1.80)
No	1797 (44.2 %)	1433 (35.3 %)		835 (20.5 %)	
Chest/breathing problem	334 (46.9 %)	208 (20.2 %)	0.75 (0.62–0.90)	170 (23.9 %)	1.18 (0.95–1.46)
No	1485 (43.7 %)	1241 (36.5 %)		675 (19.9 %)	
Diabetes	239 (48.1 %)	168 (33.8 %)	0.86 (0.70–1.07)	90 (18.1 %)	0.85 (0.65–1.11)
No	1580 (43.7 %)	1281 (35.4 %)		755 (20.9 %)	
Difficulty hearing	148 (44.6 %)	122 (36.8 %)	1.00 (0.78–1.29)	62 (18.7 %)	1.06 (0.77–1.46)
No	1671 (44.2 %)	1327 (35.1 %)		783 (20.7 %)	
Difficulty seeing	133 (43.0 %)	115 (37.2 %)	1.06 (0.82–1.38)	61 (19.7 %)	1.08 (0.78–1.50)
No	1686 (44.3 %)	1334 (35.1 %)		784 (20.6 %)	
Dyslexia	8 (24.2 %)	11 (33.3 %)	1.92 (0.77–4.81)	14 (42.4 %)	2.66 (1.08–6.56)
No	1811 (44.4 %)	1438 (35.3 %)		831 (20.4 %)	
Epilepsy	50 (46.7 %)	29 (27.1 %)	0.76 (0.48–1.22)	28 (26.2 %)	0.97 (0.59–1.59)
No	1769 (44.2 %)	1420 (35.5 %)		817 (20.4 %)	
Heart/circulatory problem	148 (44.6 %)	122 (36.8 %)	0.97 (0.83–1.13)	62 (18.7 %)	1.26 (1.05–1.52)
No	1761 (44.2 %)	1327 (35.1 %)		783 (20.7 %)	
Learning/behavioural problem	12 (40.0 %)	12 (40.0 %)	1.44 (0.64–3.24)	6 (20.0 %)	0.67 (0.24–1.86)
No	1807 (44.3 %)	1437 (35.2 %)		839 (20.6 %)	
Mental health problem	189 (42.1 %)	130 (29.0 %)	0.90 (0.70–1.17)	130 (29.0 %)	0.91 (0.69–1.19)
No	1630 (44.5 %)	1319 (36.0 %)		715 (19.5 %)	
Disability: arms, hands	202 (41.9 %)	181 (37.6 %)	1.15 (0.92–1.42)	99 (20.5 %)	1.05 (0.81–1.37)
No	1617 (44.5 %)	1268 (34.9 %)		746 (20.6 %)	
Disability: legs, feet	478 (42.7 %)	431 (38.5 %)	1.17 (1.00–1.37)	634 (21.2 %)	1.02 (0.84–1.24)
No	1341 (44.8 %)	1018 (34.0 %)		211 (18.8 %)	
Disability: neck, back	321 (44.0 %)	234 (32.1 %)	0.90 (0.75–1.08)	175 (24.0 %)	1.15 (0.93–1.42)
No	1498 (44.3 %)	1215 (35.9 %)		670 (19.8 %)	
Severe disfigurement, skin condition or allergies	41 (46.6 %)	24 (27.3 %)	0.74 (0.44–1.23)	23 (26.1 %)	1.08 (0.63–1.85)
No	1778 (44.2 %)	1425 (35.4 %)		822 (20.4 %)	
Severe stomach, liver, kidney or digestive problems	156 (44.6 %)	111 (31.7 %)	0.88 (0.68–1.14)	83 (23.7 %)	1.09 (0.82–1.46)
No	1663 (44.2 %)	1338 (35.6 %)		762 (20.3 %)	
Some other progressive disability or illness	103 (44.0 %)	86 (36.8 %)	1.05 (0.78–1.41)	45 (19.2 %)	0.92 (0.63–1.33)
No	1716 (44.2 %)	1363 (35.1 %)		800 (20.6 %)	
Some other health problem or disability	243 (43.9 %)	193 (34.9 %)	0.99 (0.81–1.22)	117 (21.2 %)	0.96 (0.75–1.23)
No	1576 (44.3 %)	1256 (35.3 %)		728 (20.5 %)	
Arthritis	551 (45.5 %)	433 (35.7 %)	0.93 (0.80–1.09)	228 (18.8 %)	1.01 (0.84–1.23)
No	1268 (43.7 %)	1016 (35.0 %)		617 (21.3 %)	

Adjusted for age and sex and ever harassment

**Table 4:** Associations between health conditions and perception on sports/leisure facilities in Scottish adults aged 16–80

	Satisfaction (*n* = 6325, 45.1 %)	Neutral (*n* = 6649, 47.4 %)	RRR (95 % CI)	Dissatisfaction (*n* = 1046, 7.5 %)	RRR (95 % CI)
Speech impairment	17 (40.5 %)	25 (59.5 %)	1.04 (0.53–2.05)	0 (0 %)	N/a
No	1359 (33.9 %)	2426 (60.4 %)		230 (5.7 %)	
Chest/breathing problem	233 (31.8 %)	449 (61.3 %)	1.16 (0.95–1.42)	51 (7.0 %)	1.51 (1.03–2.20)
No	1143 (34.4 %)	2002 (60.2 %)		179 (5.4 %)	
Diabetes	158 (32.2 %)	309 (62.9 %)	1.04 (0.82–1.31)	24 (4.9 %)	1.08 (0.67–1.76)
No	1218 (34.2 %)	2142 (60.1 %)		206 (5.8 %)	
Difficulty hearing	95 (29.0 %)	217 (66.2 %)	1.11 (0.83–1.48)	16 (4.9 %)	1.25 (0.68–2.28)
No	1281 (34.4 %)	2234 (59.9 %)		214 (5.7 %)	
Difficulty seeing	91 (28.0 %)	214 (65.9 %)	1.14 (0.85–1.53)	20 (6.2 %)	1.69 (0.99–2.89)
No	1285 (34.4 %)	2237 (59.9 %)		210 (5.6 %)	
Dyslexia	10 (32.3 %)	17 (54.8 %)	1.60 (0.65–3.96)	4 (12.9 %)	2.19 (0.64–7.51)
No	1366 (33.9 %)	2434 (60.5 %)		226 (5.6 %)	
Epilepsy	30 (29.7 %)	64 (63.4 %)	1.58 (0.95–2.63)	7 (6.9 %)	1.20 (0.48–3.02)
No	1346 (34.0 %)	2387 (60.3 %)		223 (5.6 %)	
Heart/circulatory problem	446 (32.3 %)	855 (62.0 %)	0.84 (0.71–0.99)	79 (5.7 %)	1.34 (0.95–1.89)
No	930 (34.7 %)	1596 (59.6 %)		151 (5.6 %)	
Learning/behavioural problem	9 (30.0 %)	19 (63.3 %)	2.68 (0.93–7.67)	2 (6.7 %)	1.67 (0.32–8.82)
No	1367 (34.0 %)	2432 (60.4 %)		228 (5.7 %)	
Mental health problem	188 (42.4 %)	220 (49.7 %)	1.05 (0.81–1.35)	35 (7.9 %)	0.74 (0.46–1.18)
No	1188 (32.9 %)	2231 (61.7 %)		195 (5.4 %)	
Disability: arms, hands	168 (35.2 %)	280 (58.6 %)	1.00 (0.79–1.26)	30 (6.3 %)	0.98 (0.60–1.59)
No	1208 (33.8 %)	2171 (60.7 %)		200 (5.6 %)	
Disability: legs, feet	326 (29.3 %)	724 (65.2 %)	1.27 (1.07–1.51)	61 (5.5 %)	1.15 (0.81–1.65)
No	1050 (35.6 %)	1727 (58.6 %)		169 (5.7 %)	
Disability: neck, back	217 (30.4 %)	457 (63.9 %)	1.30 (1.06–1.59)	41 (5.7 %)	0.96 (0.63–1.46)
No	1159 (34.7 %)	1994 (59.7 %)		189 (5.7 %)	
Severe disfigurement, skin condition or allergies	27 (34.6 %)	43 (55.1 %)	1.15 (0.67–1.97)	8 (10.3 %)	2.00 (0.87–4.60)
No	1349 (33.9 %)	2408 (60.5 %)		222 (5.6 %)	
Severe stomach, liver, kidney or digestive problems	123 (36.7 %)	200 (59.7 %)	1.00 (0.77–1.31)	12 (3.6 %)	0.55 (0.28–1.07)
No	1253 (33.7 %)	2251 (60.5 %)		218 (5.9 %)	
Some other progressive disability or illness	75 (31.4 %)	154 (64.4 %)	1.22 (0.87–1.70)	10 (4.2 %)	0.81 (0.38–1.72)
No	1301 (34.1 %)	2297 (60.2 %)		220 (5.8 %)	
Some other health problem or disability	185 (35.3 %)	311 (59.4 %)	0.92 (0.74–1.14)	28 (5.3 %)	0.90 (0.58–1.41)
No	1191 (33.7 %)	2140 (60.6 %)		202 (5.7 %)	
Arthritis	393 (33.1 %)	735 (62.0 %)	0.86 (0.73–1.02)	58 (4.9 %)	0.96 (0.67–1.39)
No	983 (34.2 %)	1716 (59.8 %)		172 (6.0 %)	

Adjusted for age and sex and ever harassment

**Table 5:** Associations between health conditions and perception on library facilities in Scottish adults aged 16–80

	Satisfaction (*n* = 7769, 55.4 %)	Neutral (*n* = 5883, 42.0 %)	RRR (95 % CI)	Dissatisfaction (*n* = 368, 2.6 %)	RRR (95 % CI)
Speech impairment	13 (31.0 %)	29 (69.1 %)	3.34 (1.61–6.93)	0 (0 %)	N/a
No	2114 (52.7 %)	1793 (44.7 %)		108 (2.7 %)	
Chest/breathing problem	361 (49.3 %)	353 (48.2 %)	1.11 (0.93–1.34)	19 (2.6 %)	0.89 (0.51–1.57)
No	1766 (53.1 %)	1469 (44.2 %)		89 (2.7 %)	
Diabetes	259 (52.8 %)	218 (44.4 %)	0.99 (0.80–1.23)	14 (2.9 %)	0.70 (0.33–1.47)
No	1868 (52.4 %)	1604 (45.0 %)		94 (2.6 %)	
Difficulty hearing	157 (47.9 %)	166 (50.6 %)	1.14 (0.88–1.48)	5 (1.5 %)	0.69 (0.27–1.75)
No	1970 (52.8 %)	1656 (44.4 %)		103 (2.8 %)	
Difficulty seeing	141 (43.4 %)	169 (52.0 %)	1.44 (1.10–1.88)	15 (4.6 %)	2.54 (1.38–4.66)
No	1986 (53.2 %)	1653 (24.3 %)		93 (2.5 %)	
Dyslexia	11 (35.5 %)	17 (54.8 %)	1.79 (0.78–4.14)	3 (9.7 %)	2.95 (0.62–14.03)
No	2166 (52.6 %)	1805 (44.8 %)		105 (2.6 %)	
Epilepsy	42 (41.6 %)	57 (56.4 %)	1.79 (1.13–2.86)	2 (2.0 %)	0.98 (0.23–4.19)
No	2085 (52.7 %)	1765 (44.6 %)		106 (2.7 %)	
Heart/circulatory problem	738 (53.5 %)	602 (43.6 %)	0.87 (0.74–1.01)	40 (2.9 %)	1.35 (0.87–2.10)
No	1389 (51.9 %)	1220 (45.6 %)		68 (2.5 %)	
Learning/behavioural problem	12 (40.0 %)	15 (50.0 %)	1.70 (0.66–4.34)	3 (10.0 %)	3.91 (0.79–19.39)
No	2115 (52.5 %)	1807 (44.9 %)		105 (2.6 %)	
Mental health problem	240 (54.2 %)	187 (42.2 %)	1.03 (0.81–1.33)	16 (3.6 %)	1.07 (0.57–2.01)
No	1887 (52.2 %)	1635 (45.2 %)		92 (2.6 %)	
Disability: arms, hands	1872 (52.3 %)	1612 (45.0 %)	1.03 (0.83–1.28)	95 (2.7 %)	1.06 (0.57–1.98)
No	255 (53.4 %)	210 (43.9 %)		13 (2.7 %)	
Disability: legs, feet	559 (50.3 %)	526 (47.3 %)	1.13 (0.97–1.33)	26 (2.3 %)	0.83 (0.51–1.36)
No	1568 (53.2 %)	1296 (44.0 %)		82 (2.8 %)	
Disability: neck, back	375 (52.5 %)	315 (44.1 %)	0.98 (0.82–1.18)	25 (3.5 %)	1.27 (0.78–2.08)
No	1752 (52.4 %)	1507 (45.1 %)		83 (2.5 %)	
Severe disfigurement, skin condition or allergies	47 (60.3 %)	30 (38.5 %)	0.80 (0.49–1.31)	1 (1.3 %)	0.40 (0.05–2.93)
No	2080 (52.3 %)	1792 (45.0 %)		107 (2.7 %)	
Severe stomach, liver, kidney or digestive problems	178 (53.1 %)	147 (43.9 %)	1.02 (0.79–1.32)	10 (3.0 %)	1.19 (0.60–2.35)
No	1949 (52.4 %)	1675 (45.0 %)		98 (2.6 %)	
Some other progressive disability or illness	130 (54.4 %)	101 (42.3 %)	0.99 (0.72–1.35)	8 (3.4 %)	1.43 (0.67–3.06)
No	1997 (52.3 %)	1721 (45.1 %)		100 (2.6 %)	
Some other health problem or disability	1850 (52.4 %)	1587 (44.9 %)	1.11 (0.90–1.36)	96 (2.7 %)	0.86 (0.46–1.60)
No	277 (52.9 %)	235 (44.9 %)		12 (2.3 %)	
Arthritis	1495 (52.1 %)	1297 (45.2 %)	0.88 (0.75–1.03)	79 (2.8 %)	0.83 (0.51–1.33)
No	632 (53.3 %)	525 (44.3 %)		29 (2.5 %)	

Adjusted for age and sex and ever harassment

**Table 6:** Associations between health conditions and perception on museum/gallery facilities in Scottish adults aged 16–80

	Satisfaction (*n* = 5629, 40.2 %)	Neutral (*n* = 7906, 56.4 %)	RRR (95 % CI)	Dissatisfaction (*n* = 485, 3.5 %)	RRR (95 % CI)
Speech impairment	11 (26.2 %)	30 (71.4 %)	1.95 (0.89–4.31)	1 (2.4 %)	1.26 (0.16–10.19)
No	1347 (33.6 %)	2547 (63.4 %)		121 (3.0 %)	
Chest/breathing problem	245 (33.4 %)	467 (63.7 %)	0.95 (0.79–1.16)	21 (2.9 %)	0.90 (0.52–1.55)
No	1113 (33.5 %)	2110 (63.5 %)		101 (3.5 %)	
Diabetes	181 (36.9 %)	294 (59.9 %)	0.80 (0.64–0.99)	16 (3.3 %)	0.75 (0.39–1.45)
No	1177 (33.0 %)	2283 (64.0 %)		106 (3.0 %)	
Difficulty hearing	97 (29.6 %)	221 (67.4 %)	1.11 (0.84–1.47)	10 (3.1 %)	1.26 (0.60–2.61)
No	1261 (33.8 %)	2356 (63.2 %)		112 (3.0 %)	
Difficulty seeing	91 (28.0 %)	220 (67.7 %)	1.35 (1.01–1.81)	14 (4.3 %)	2.23 (1.18–4.21)
No	1267 (34.0 %)	2357 (63.2 %)		108 (2.9 %)	
Dyslexia	14 (45.2 %)	15 (48.4 %)	0.49 (0.21–1.13)	2 (6.5 %)	1.52 (0.33–7.00)
No	1344 (33.4 %)	2562 (63.2 %)		120 (3.0 %)	
Epilepsy	33 (32.7 %)	67 (66.3 %)	1.27 (0.78–2.07)	1 (1.0 %)	N/a
No	1325 (33.5 %)	2510 (63.5 %)		121 (3.1 %)	
Heart/circulatory problem	444 (32.2 %)	888 (64.4 %)	1.02 (0.87–1.20)	48 (3.5 %)	1.31 (0.85–2.03)
No	914 (34.1 %)	1689 (63.1 %)		74 (2.8 %)	
Learning/behavioural problem	3 (10.0 %)	27 (90.0 %)	6.02 (1.39–26.13)	0 (0 %)	N/a
No	1355 (33.7 %)	2550 (63.3 %)		122 (3.0 %)	
Mental health problem	153 (34.5 %)	279 (63.0 %)	1.11 (0.86–1.43)	11 (2.5 %)	0.61 (0.29–1.30)
No	1205 (33.3 %)	2298 (63.6 %)		111 (3.1 %)	
Disability: arms, hands	153 (32.0 %)	303 (63.4 %)	1.18 (0.93–1.49)	22 (4.6 %)	1.98 (1.16–3.38)
No	1205 (33.7 %)	2274 (63.5 %)		100 (2.8 %)	
Disability: legs, feet	333 (30.0 %)	740 (66.6 %)	1.22 (1.03–1.44)	38 (3.4 %)	1.71 (1.11–2.63)
No	1025 (34.8 %)	1837 (62.4 %)		84 (2.9 %)	
Disability: neck, back	232 (32.5 %)	456 (63.8 %)	1.14 (0.93–1.38)	27 (3.8 %)	1.49 (0.92–2.43)
No	1126 (33.7 %)	2121 (63.5 %)		95 (2.8 %)	
Severe disfigurement, skin condition or allergies	30 (38.5 %)	46 (59.0 %)	0.94 (0.57–1.56)	2 (2.6 %)	0.83 (0.19–3.59)
No	1328 (33.4 %)	2531 (63.6 %)		120 (3.0 %)	
Severe stomach, liver, kidney or digestive problems	116 (34.6 %)	212 (63.3 %)	1.02 (0.78–1.33)	7 (2.1 %)	0.54 (0.21–1.36)
No	1242 (33.4 %)	2365 (63.5 %)		115 (3.1 %)	
Some other progressive disability or illness	87 (36.4 %)	147 (61.5 %)	0.87 (0.63–1.19)	5 (2.1 %)	0.78 (0.31–1.98)
No	1271 (33.3 %)	2430 (63.7 %)		117 (3.1 %)	
Some other health problem or disability	173 (33.0 %)	336 (64.1 %)	1.12 (0.90–1.39)	15 (2.9 %)	1.12 (0.63–2.00)
No	1185 (33.5 %)	2241 (63.4 %)		107 (3.0 %)	
Arthritis	399 (33.6 %)	753 (63.5 %)	0.96 (0.82–1.14)	34 (2.9 %)	1.00 (0.63–1.59)
No	959 (33.4 %)	1824 (63.5 %)		88 (3.1 %)	

Adjusted for age and sex and ever harassment

**Table 7:** Associations between health conditions and perception on theatre facilities in Scottish adults aged 16–80

	Satisfaction (*n* = 5789, 41.3 %)	Neutral (*n* = 7630, 54.4 %)	RRR (95 % CI)	Dissatisfaction (*n* = 601, 4.3 %)	RRR (95 % CI)
Speech impairment	12 (28.6 %)	30 (71.4 %)	1.71 (0.80–3.65)	0 (0 %)	N/a
No	1383 (34.5 %)	2506 (62.4 %)		126 (3.1 %)	
Chest/breathing problem	232 (31.7 %)	480 (65.5 %)	1.17 (0.96–1.42)	21 (2.9 %)	1.02 (0.60–1.73)
No	1163 (35.0 %)	2056 (61.9 %)		105 (3.2 %)	
Diabetes	187 (38.1 %)	290 (59.1 %)	0.84 (0.67–1.05)	14 (2.9 %)	0.67 (0.34–1.32)
No	1208 (33.9 %)	2246 (63.0 %)		112 (3.1 %)	
Difficulty hearing	103 (31.4 %)	217 (66.2 %)	1.05 (0.80–1.38)	8 (2.4 %)	0.84 (0.38–1.88)
No	1292 (34.7 %)	2319 (62.2 %)		118 (3.2 %)	
Difficulty seeing	105 (32.3 %)	208 (64.0 %)	1.04 (0.79–1.36)	12 (3.7 %)	1.39 (0.71–2.70)
No	1290 (34.6 %)	2328 (62.4 %)		114 (3.1 %)	
Dyslexia	8 (25.8 %)	18 (58.1 %)	1.07 (0.42–2.71)	5 (16.1 %)	7.31 (2.22–24.09)
No	1387 (34.5 %)	2518 (62.5 %)		121 (3.0 %)	
Epilepsy	27 (26.7 %)	72 (71.3 %)	1.80 (1.06–3.05)	2 (2.0 %)	0.50 (0.07–3.88)
No	1368 (34.6 %)	2464 (62.3 %)		124 (3.1 %)	
Heart/circulatory problem	492 (35.7 %)	846 (81.3 %)	0.83 (0.71–0.97)	42 (3.0 %)	0.85 (0.55–1.32)
No	903 (33.7 %)	1690 (63.1 %)		84 (3.1 %)	
Learning/behavioural problem	2 (6.7 %)	28 (93.3 %)	10.90 (1.45–81.82)	0 (0 %)	N/a
No	1393 (34.6 %)	2508 (62.3 %)		126 (3.1 %)	
Mental health problem	140 (31.6 %)	290 (65.5 %)	1.26 (0.97–1.64)	13 (2.9 %)	0.97 (0.49–1.91)
No	1255 (34.7 %)	2246 (62.2 %)		113 (3.1 %)	
Disability: arms, hands	148 (31.0 %)	312 (65.3 %)	1.39 (1.09–1.76)	18 (3.8 %)	1.52 (0.85–2.71)
No	1247 (34.8 %)	2224 (62.1 %)		108 (3.0 %)	
Disability: legs, feet	349 (31.4 %)	732 (65.9 %)	1.30 (1.10–1.54)	30 (2.7 %)	1.04 (0.66–1.65)
No	1046 (35.5 %)	1804 (61.2 %)		96 (3.3 %)	
Disability: neck, back	232 (32.5 %)	460 (64.3 %)	1.28 (1.05–1.56)	23 (3.2 %)	1.31 (0.80–2.15)
No	1163 (34.8 %)	2076 (62.1 %)		103 (3.1 %)	
Severe disfigurement, skin condition or allergies	29 (37.2 %)	47 (60.3 %)	0.95 (0.58–1.58)	2 (2.6 %)	0.80 (0.19–3.45)
No	1366 (34.3 %)	2489 (62.6 %)		124 (3.1 %)	
Severe stomach, liver, kidney or digestive problems	120 (35.8 %)	208 (62.1 %)	0.98 (0.75–1.27)	7 (2.1 %)	0.60 (0.26–1.41)
No	1275 (34.3 %)	2328 (62.6 %)		119 (3.2 %)	
Some other progressive disability or illness	82 (34.3 %)	148 (61.9 %)	1.02 (0.74–1.40)	9 (3.8 %)	1.34 (0.62–2.88)
No	1313 (34.4 %)	2388 (62.6 %)		117 (3.1 %)	
Some other health problem or disability	185 (35.3 %)	324 (61.8 %)	0.95 (0.77–1.17)	15 (2.9 %)	0.87 (0.49–1.57)
No	1210 (34.3 %)	2212 (62.2 %)		111 (3.1 %)	
Arthritis	435 (36.7 %)	715 (60.3 %)	0.88 (0.74–1.03)	36 (3.0 %)	0.93 (0.60–1.46)
No	960 (33.4 %)	1821 (63.4 %)		90 (3.1 %)	

Adjusted for age and sex and ever harassment

**Table 8:** Associations between health conditions and perception on parks and open space in Scottish adults aged 16–80

	Satisfaction (*n* = 9014, 64.3 %)	Neutral (*n* = 4073, 29.1 %)	RRR (95 % CI)	Dissatisfaction (*n* = 933, 6.7 %)	RRR (95 % CI)
Speech impairment	21 (50.0 %)	19 (45.2 %)	1.52 (0.77–3.00)	2 (4.8 %)	1.00 (0.23–4.36)
No	2225 (55.4 %)	1549 (38.6 %)		241 (6.0 %)	
Chest/breathing problem	377 (51.4 %)	1261 (37.9 %)	1.14 (0.94–1.38)	49 (6.7 %)	1.26 (0.88–1.82)
No	1869 (56.2 %)	3077 (41.9 %)		194 (5.8 %)	
Diabetes	2724 (55.8 %)	189 (38.5 %)	0.95 (0.76–1.20)	28 (5.7 %)	1.02 (0.64–1.62)
No	1972 (55.3 %)	1379 (38.7 %)		215 (6.0 %)	
Difficulty hearing	159 (48.5 %)	152 (46.3 %)	1.08 (0.82–1.41)	17 (5.2 %)	1.18 (0.68–2.04)
No	2087 (56.0 %)	1416 (38.0 %)		226 (6.1 %)	
Difficulty seeing	157 (48.3 %)	146 (44.9 %)	*1.30 (0.99–1.70)*	22 (6.8 %)	1.41 (0.83–2.37)
No	2089 (56.0 %)	1422 (38.1 %)		221 (5.9 %)	
Dyslexia	20 (64.5 %)	10 (32.3 %)	0.97 (0.40–2.40)	1 (3.2 %)	0.43 (0.06–3.28)
No	2226 (55.3 %)	1558 (38.7 %)		242 (6.0 %)	
Epilepsy	58 (57.4 %)	35 (34.7 %)	1.19 (0.73–1.94)	8 (7.9 %)	0.89 (0.34–2.28)
No	2188 (55.3 %)	1533 (38.8 %)		235 (5.9 %)	
Heart/circulatory problem	749 (54.3 %)	539 (39.1 %)	0.87 (0.74–1.02)	92 (6.7 %)	1.44 (1.05–1.98)
No	1497 (55.9 %)	1029 (38.4 %)		151 (5.6 %)	
Learning/behavioural problem	14 (46.7 %)	13 (43.3 %)	1.55 (0.59–4.08)	3 (10.0 %)	1.41 (0.3–6.53)
No	2232 (55.4 %)	1555 (38.6 %)		240 (6.0 %)	
Mental health problem	263 (59.4 %)	136 (30.7 %)	1.07 (0.82–1.41)	44 (9.9 %)	1.24 (0.81–1.90)
No	1983 (54.9 %)	1432 (39.6 %)		199 (5.5 %)	
Disability: arms, hands	258 (54.0 %)	182 (38.1 %)	1.07 (0.84–1.34)	38 (8.0 %)	1.47 (0.98–2.22)
No	1988 (55.6 %)	1386 (38.7 %)		205 (5.7 %)	
Disability: legs, feet	554 (59.9 %)	490 (44.1 %)	1.30 (1.10–1.53)	67 (6.0 %)	1.23 (0.89–1.71)
No	1692 (57.4 %)	1078 (36.6 %)		176 (6.0 %)	
Disability: neck, back	384 (53.7 %)	288 (40.3 %)	1.21 (1.00–1.47)	43 (6.0 %)	1.07 (0.74–1.56)
No	1862 (55.7 %)	1280 (38.3 %)		200 (6.0 %)	
Severe disfigurement, skin condition or allergies	42 (53.9 %)	29 (37.2 %)	1.11 (0.66–1.87)	7 (9.0 %)	1.63 (0.71–3.73)
No	2204 (55.4 %)	1539 (38.7 %)		236 (5.9 %)	
Severe stomach, liver, kidney or digestive problems	173 (51.6 %)	132 (39.4 %)	1.20 (0.92–1.58)	30 (9.0 %)	1.71 (1.09–2.67)
No	2073 (55.7 %)	1436 (38.6 %)		213 (5.7 %)	
Some other progressive disability or illness	127 (53.1 %)	98 (41.0 %)	1.19 (0.87–1.63)	14 (5.9 %)	0.68 (0.32–1.43)
No	2119 (55.5 %)	1470 (38.5 %)		229 (6.0 %)	
Some other health problem or disability	300 (57.3 %)	195 (37.2 %)	0.90 (0.73–1.12)	29 (5.5 %)	0.79 (0.51–1.23)
No	1946 (55.1 %)	1373 (38.9 %)		214 (6.1 %)	
Arthritis	628 (53.0 %)	474 (40.0 %)	0.93 (0.79–1.10)	84 (7.1 %)	1.34 (0.97–1.85)
No	1618 (56.4 %)	1094 (38.1 %)		159 (5.5 %)	

Adjusted for age and sex and ever harassment

**Table 9:** Associations between health conditions and perception on refuse collection in Scottish adults aged 16–80

	Satisfaction (*n* = 11,332, 80.8 %)	Neutral (*n* = 938, 6.7 %)	RRR (95 % CI)	Dissatisfaction (*n* = 1748, 12.5 %)	RRR (95 % CI)
Speech impairment	34 (81.0 %)	4 (9.5 %)	1.82 (0.63–5.24)	4 (9.5 %)	1.17 (0.40–3.39)
No	3359 (83.7 %)	258 (6.4 %)		398 (9.9 %)	
Chest/breathing problem	608 (83.0 %)	35 (4.8 %)	0.79 (0.53–1.19)	90 (12.3 %)	1.46 (1.09–1.94)
No	2785 (83.8 %)	227 (6.8 %)		312 (9.4 %)	
Diabetes	409 (83.3 %)	28 (5.7 %)	1.00 (0.63–1.57)	54 (11.0 %)	1.27 (0.88–1.81)
No	2984 (83.7 %)	234 (6.6 %)		348 (9.8 %)	
Difficulty hearing	283 (86.3 %)	17 (5.2 %)	0.97 (0.55–1.71)	28 (8.5 %)	0.93 (0.57–1.53)
No	3110 (83.4 %)	245 (6.6 %)		374 (10.0 %)	
Difficulty seeing	262 (80.6 %)	20 (6.2 %)	1.00 (0.57–1.76)	43 (13.2 %)	1.73 (1.16–2.58)
No	3131 (83.9 %)	242 (6.5 %)		359 (9.6 %)	
Dyslexia	26 (83.9 %)	0 (0 %)	N/a	5 (16.1 %)	0.39 (0.09–1.69)
No	3367 (83.6 %)	262 (6.5 %)		397 (9.9 %)	
Epilepsy	80 (79.2 %)	7 (6.9 %)	0.98 (0.41–2.31)	14 (13.9 %)	0.64 (0.29–1.44)
No	3313 (83.8 %)	255 (6.5 %)		388 (9.8 %)	
Heart/circulatory problem	1186 (85.9 %)	73 (5.3 %)	0.92 (0.66–1.27)	121 (8.8 %)	1.10 (0.83–1.44)
No	2207 (82.4 %)	189 (7.1 %)		281 (10.5 %)	
Learning/behavioural problem	25 (83.3 %)	3 (10.0 %)	1.06 (0.24–4.69)	2 (6.7 %)	0.27 (0.04–2.07)
No	3368 (83.6 %)	259 (6.4 %)		400 (9.9 %)	
Mental health problem	334 (75.4 %)	38 (8.6 %)	1.08 (0.69–1.69)	71 (16.0 %)	1.00 (0.70–1.42)
No	3059 (84.6 %)	224 (6.2 %)		331 (9.2 %)	
Disability: arms, hands	388 (81.2 %)	41 (8.6 %)	1.34 (0.90–2.02)	49 (10.3 %)	1.05 (0.73–1.52)
No	3005 (84.0 %)	221 (6.2 %)		353 (9.9 %)	
Disability: legs, feet	931 (83.8 %)	82 (7.4 %)	1.18 (0.86–1.62)	98 (8.8 %)	0.95 (0.72–1.25)
No	2462 (83.6 %)	180 (6.1 %)		304 (10.3 %)	
Disability: neck, back	598 (83.6 %)	48 (6.7 %)	0.96 (0.66–1.40)	69 (9.7 %)	0.93 (0.68–1.28)
No	2795 (83.6 %)	214 (6.4 %)		333 (10.0 %)	
Severe disfigurement, skin condition or allergies	65 (83.3 %)	6 (7.7 %)	0.82 (0.29–2.28)	7 (9.0 %)	0.75 (0.32–1.78)
No	3328 (83.6 %)	256 (6.4 %)		395 (9.9 %)	
Severe stomach, liver, kidney or digestive problems	283 (84.5 %)	15 (4.5 %)	0.78 (0.45–1.37)	37 (11.0 %)	1.11 (0.74–1.66)
No	3110 (83.6 %)	247 (6.6 %)		365 (9.8 %)	
Some other progressive disability or illness	192 (80.3 %)	16 (6.7 %)	0.88 (0.46–1.70)	31 (13.0 %)	1.15 (0.70–1.88)
No	3201 (83.8 %)	246 (6.4 %)		371 (9.7 %)	
Some other health problem or disability	432 (82.4 %)	34 (6.5 %)	0.98 (0.65–1.49)	58 (11.1 %)	0.99 (0.71–1.40)
No	2961 (83.8 %)	228 (6.5 %)		344 (9.7 %)	
Arthritis	987 (83.2 %)	78 (6.6 %)	1.27 (0.92–1.75)	121 (10.2 %)	1.32 (1.01–1.74)
No	2406 (83.8 %)	184 (6.4 %)		281 (9.8 %)	

Adjusted for age and sex and ever harassment

**Table 10:** Associations between health conditions and perception on local schools in Scottish adults aged 16–80

	Satisfaction (*n* = 6292, 44.9 %)	Neutral (*n* = 7400, 52.8 %)	RRR (95 % CI)	Dissatisfaction (*n* = 325, 2.3 %)	RRR (95 % CI)
Speech impairment	11 (26.2 %)	31 (73.8 %)	1.82 (0.87–3.80)	0 (0 %)	N/a
No	1493 (37.2 %)	2433 (60.6 %)		88 (2.2 %)	
Chest/breathing problem	263 (35.9 %)	453 (61.8 %)	1.00 (0.83–1.21)	17 (2.3 %)	1.11 (0.60–2.02)
No	1241 (37.4 %)	2011 (60.5 %)		71 (2.1 %)	
Diabetes	183 (37.3 %)	302 (61.5 %)	0.87 (0.69–1.08)	6 (1.2 %)	0.47 (0.17–1.31)
No	1321 (37.1 %)	2162 (60.7 %)		82 (2.3 %)	
Difficulty hearing	101 (30.8 %)	221 (67.4 %)	1.17 (0.88–1.55)	6 (1.8 %)	1.43 (0.59–3.47)
No	1403 (37.6 %)	2243 (60.2 %)		82 (2.2 %)	
Difficulty seeing	90 (27.7 %)	229 (70.5 %)	1.49 (1.11–1.98)	6 (1.9 %)	0.98 (0.35–2.79)
No	1414 (37.9 %)	2235 (59.9 %)		82 (2.2 %)	
Dyslexia	6 (19.4 %)	23 (74.2 %)	3.91 (1.42–10.77)	2 (6.5 %)	5.37 (0.98–29.40)
No	1498 (37.2 %)	2441 (60.7 %)		86 (2.1 %)	
Epilepsy	35 (34.7 %)	60 (59.4 %)	1.26 (0.77–2.05)	6 (5.9 %)	2.59 (0.95–7.08)
No	1469 (37.1 %)	2404 (60.8 %)		82 (2.1 %)	
Heart/circulatory problem	503 (36.5 %)	855 (62.0 %)	0.82 (0.70–0.96)	22 (1.6 %)	0.79 (0.45–1.38)
No	101 (37.4 %)	1609 (60.1 %)		66 (2.5 %)	
Learning/behavioural problem	10 (33.3 %)	20 (66.7 %)	1.53 (0.61–3.83)	0 (0 %)	N/a
No	1494 (37.1 %)	2444 (60.7 %)		88 (2.2 %)	
Mental health problem	177 (40.0 %)	252 (56.9 %)	1.40 (1.09–1.81)	14 (3.2 %)	0.70 (0.34–1.36)
No	1327 (36.7 %)	2212 (61.2 %)		74 (2.1 %)	
Disability: arms, hands	175 (36.6 %)	288 (60.3 %)	1.05 (0.83–1.32)	15 (3.1 %)	1.81 (0.98–3.35)
No	1329 (37.1 %)	2176 (60.8 %)		73 (2.0 %)	
Disability: legs, feet	386 (34.7 %)	694 (62.5 %)	1.04 (0.88–1.23)	31 (2.8 %)	2.03 (1.24–3.32)
No	1118 (38.0 %)	1770 (60.1 %)		57 (1.9 %)	
Disability: neck, back	279 (39.0 %)	411 (57.5 %)	0.94 (0.78–1.14)	25 (3.5 %)	1.91 (0.95–2.73)
No	1225 (36.7 %)	2053 (61.5 %)		63 (1.9 %)	
Severe disfigurement, skin condition or allergies	28 (35.9 %)	44 (56.4 %)	1.06 (0.63–1.78)	6 (7.7 %)	3.98 (1.55–10.23)
No	1476 (37.1 %)	2420 (60.8 %)		82 (2.1 %)	
Severe stomach, liver, kidney or digestive problems	128 (38.2 %)	197 (58.8 %)	0.96 (0.74–1.25)	10 (3.0 %)	1.36 (0.65–2.83)
No	1376 (37.0 %)	2267 (60.9 %)		78 (2.1 %)	
Some other progressive disability or illness	84 (35.2 %)	149 (62.3 %)	1.11 (0.81–1.54)	6 (2.5 %)	1.16 (0.45–3.02)
No	1420 (37.2 %)	2315 (60.7 %)		82 (2.2 %)	
Some other health problem or disability	212 (40.5 %)	296 (56.6 %)	0.80 (0.65–0.99)	15 (2.9 %)	1.07 (0.57–2.00)
No	1292 (36.6 %)	2168 (61.4 %)		73 (2.1 %)	
Arthritis	417 (35.2 %)	742 (62.6 %)	0.95 (0.80–1.11)	26 (2.2 %)	1.30 (0.76–2.21)
No	1087 (37.9 %)	1722 (60.0 %)		62 (2.2 %)	

Adjusted for age and sex and ever harassment

**Table 11:** Associations between health conditions and perception on public transportation in Scottish adults aged 16–80

	Satisfaction (*n* = 8697, 62.1 %)	Neutral (*n* = 3561, 25.4 %)	RRR (95 % CI)	Dissatisfaction (*n* = 1759, 12.6 %)	RRR (95 % CI)
Speech impairment	26 (61.9 %)	13 (31.0 %)	1.56 (0.78–3.14)	3 (7.1 %)	0.71 (0.21–2.40)
No	2508 (62.5 %)	1027 (25.6 %)		479 (11.9 %)	
Chest/breathing problem	463 (63.2 %)	185 (25.2 %)	0.93 (0.75–1.15)	85 (11.6 %)	0.92 (0.69–1.23)
No	2071 (62.3 %)	855 (25.7 %)		397 (12.0 %)	
Diabetes	310 (63.1 %)	128 (26.1 %)	1.01 (0.79–1.30)	53 (10.8 %)	0.94 (0.66–1.33)
No	2224 (62.4 %)	912 (25.6 %)		429 (12.0 %)	
Difficulty hearing	200 (61.0 %)	94 (28.7 %)	1.07 (0.80–1.44)	34 (10.4 %)	0.92 (0.59–1.43)
No	2334 (62.6 %)	946 (25.4 %)		448 (12.0 %)	
Difficulty seeing	198 (60.9 %)	91 (28.0 %)	1.14 (0.85–1.52)	36 (11.1 %)	0.98 (0.64–1.51)
No	2336 (62.6 %)	949 (25.4 %)		446 (12.0 %)	
Dyslexia	13 (41.9 %)	7 (22.6 %)	1.35 (0.46–3.92)	11 (35.5 %)	3.13 (1.27–7.73)
No	2521 (62.6 %)	1033 (25.7 %)		471 (11.7 %)	
Epilepsy	59 (58.4 %)	29 (28.7 %)	1.39 (0.83–2.33)	13 (12.9 %)	0.93 (0.46–1.88)
No	2475 (62.6 %)	1011 (25.6 %)		469 (11.9 %)	
Heart/circulatory problem	878 (63.6 %)	360 (26.1 %)	0.96 (0.81–1.14)	142 (10.3 %)	0.86 (0.67–1.11)
No	1656 (61.9 %)	680 (25.4 %)		340 (12.7 %)	
Learning/behavioural problem	19 (63.3 %)	7 (23.3 %)	1.27 (0.44–3.64)	4 (13.3 %)	0.92 (0.26–3.33)
No	2515 (62.5 %)	1033 (25.7 %)		478 (11.9 %)	
Mental health problem	284 (64.1 %)	96 (21.7 %)	1.10 (0.82–1.48)	63 (14.2 %)	0.81 (0.57–1.15)
No	2250 (62.3 %)	944 (26.1 %)		419 (11.6 %)	
Disability: arms, hands	268 (56.1 %)	163 (34.1 %)	1.55 (1.22–1.97)	47 (9.8 %)	0.82 (0.56–1.20)
No	2266 (63.3 %)	877 (24.5 %)		435 (12.2 %)	
Disability: legs, feet	612 (55.1 %)	381 (34.3 %)	1.74 (1.46–2.08)	118 (10.6 %)	1.02 (0.79–1.32)
No	1922 (65.3 %)	659 (22.4 %)		364 (12.4 %)	
Disability: neck, back	417 (58.3 %)	201 (28.1 %)	1.24 (1.01–1.53)	97 (13.6 %)	1.14 (0.86–1.51)
No	2117 (63.4 %)	839 (25.1 %)		385 (11.5 %)	
Severe disfigurement, skin condition or allergies	52 (66.7 %)	19 (24.4 %)	0.95 (0.54–1.67)	7 (9.0 %)	0.73 (0.32–1.63)
No	2482 (62.4 %)	1021 (25.7 %)		475 (11.9 %)	
Severe stomach, liver, kidney or digestive problems	206 (61.5 %)	92 (27.5 %)	1.14 (0.86–1.52)	37 (11.0 %)	0.86 (0.57–1.29)
No	2328 (62.6 %)	948 (25.5 %)		445 (12.0 %)	
Some other progressive disability or illness	131 (54.8 %)	80 (33.5 %)	1.47 (1.05–2.06)	28 (11.7 %)	1.25 (0.79–1.99)
No	2403 (63.0 %)	960 (25.2 %)		454 (11.9 %)	
Some other health problem or disability	318 (60.8 %)	137 (26.2 %)	0.98 (0.77–1.25)	68 (13.0 %)	0.99 (0.72–1.35)
No	2216 (62.7 %)	903 (25.6 %)		414 (11.7 %)	
Arthritis	721 (60.8 %)	340 (28.7 %)	1.09 (0.92–1.31)	124 (10.5 %)	0.98 (0.76–1.27)
No	1813 (63.2 %)	700 (24.4 %)		358 (12.5 %)	

Adjusted for age and sex and ever harassment

**Table 12:** Associations between health conditions and perception on street cleaning in Scottish adults aged 16–80

	Satisfaction (*n* = 9650, 68.8 %)	Neutral (*n* = 2076, 14.8 %)	RRR (95 % CI)	Dissatisfaction (*n* = 2291, 16.3 %)	RRR (95 % CI)
Speech impairment	28 (66.7 %)	7 (16.7 %)	1.24 (0.51–3.05)	7 (16.7 %)	0.93 (0.38–2.30)
No	2761 (68.8 %)	553 (13.8 %)		700 (17.4 %)	
Chest/breathing problem	523 (71.4 %)	84 (11.5 %)	0.72 (0.54–0.96)	126 (17.2 %)	0.99 (0.78–1.26)
No	2266 (68.2 %)	476 (14.3 %)		581 (17.5 %)	
Diabetes	332 (67.6 %)	69 (14.1 %)	1.04 (0.76–1.42)	90 (18.3 %)	1.05 (0.78–1.39)
No	2457 (68.9 %)	491 (13.8 %)		617 (17.3 %)	
Difficulty hearing	222 (67.7 %)	51 (15.6 %)	1.14 (0.79–1.65)	55 (16.8 %)	1.11 (0.79–1.57)
No	2567 (68.9 %)	509 (13.7 %)		652 (17.5 %)	
Difficulty seeing	221 (68.0 %)	47 (14.5 %)	1.14 (0.79–1.64)	57 (17.5 %)	1.04 (0.73–1.47)
No	2568 (68.8 %)	513 (13.8 %)		650 (17.4 %)	
Dyslexia	19 (61.3 %)	3 (9.7 %)	0.66 (0.15–2.90)	9 (29.0 %)	1.52 (0.61–3.77)
No	2770 (68.8 %)	557 (13.8 %)		698 (17.3 %)	
Epilepsy	70 (69.3 %)	16 (15.8 %)	1.24 (0.67–2.31)	15 (14.9 %)	0.66 (0.33–1.32)
No	2719 (68.8 %)	544 (13.8 %)		692 (17.5 %)	
Heart/circulatory problem	939 (68.1 %)	182 (13.2 %)	0.86 (0.68–1.08)	259 (18.8 %)	1.31 (1.07–1.60)
No	1850 (69.1 %)	378 (14.1 %)		448 (16.7 %)	
Learning/behavioural problem	19 (63.3 %)	6 (20.0 %)	2.26 (0.79–6.51)	5 (16.7 %)	0.86 (0.24–3.11)
No	2770 (68.8 %)	554 (13.8 %)		702 (17.4 %)	
Mental health problem	302 (68.2 %)	54 (12.2 %)	0.81 (0.55–1.19)	87 (19.6 %)	0.91 (0.66–1.24)
No	2487 (68.8 %)	506 (14.0 %)		620 (17.2 %)	
Disability: arms, hands	312 (65.3 %)	74 (15.5 %)	1.17 (0.86–1.60)	92 (19.3 %)	1.15 (0.87–1.53)
No	2477 (69.2 %)	486 (13.6 %)		615 (17.2 %)	
Disability: legs, feet	756 (68.1 %)	172 (15.5 %)	1.23 (0.98–1.54)	183 (16.5 %)	0.98 (0.80–1.22)
No	2033 (69.0 %)	388 (13.2 %)		524 (17.8 %)	
Disability: neck, back	474 (66.3 %)	108 (15.1 %)	1.20 (0.92–1.55)	133 (18.6 %)	1.06 (0.83–1.34)
No	2315 (69.3 %)	452 (13.5 %)		574 (17.2 %)	
Severe disfigurement, skin condition or allergies	58 (74.4 %)	11 (14.1 %)	1.01 (0.51–2.01)	9 (11.5 %)	0.59 (0.58–1.26)
No	2731 (68.7 %)	549 (13.8 %)		698 (17.6 %)	
Severe stomach, liver, kidney or digestive problems	224 (66.9 %)	56 (16.7 %)	1.40 (1.00–1.96)	55 (16.4 %)	0.97 (0.69–1.37)
No	2565 (68.9 %)	504 (13.5 %)		562 (17.5 %)	
Some other progressive disability or illness	163 (68.2 %)	34 (14.2 %)	1.07 (0.68–1.66)	42 (17.6 %)	1.05 (0.70–1.57)
No	2626 (68.8 %)	526 (13.8 %)		665 (17.4 %)	
Some other health problem or disability	357 (68.3 %)	71 (13.6 %)	0.97 (0.71–1.31)	95 (18.2 %)	1.08 (0.83–1.41)
No	2432 (68.8 %)	489 (13.8 %)		612 (17.3 %)	
Arthritis	799 (67.4 %)	161 (13.6 %)	0.98 (0.77–1.23)	225 (19.0 %)	1.11 (0.90–1.37)
No	1990 (69.3 %)	399 (13.9 %)		482 (16.8 %)	

Adjusted for age and sex and ever harassment

In addition, people with vision (RRR, 1.80 (95 % CI, 1.02–3.19); *P* = 0.043) and legs (RRR, 1.69 (95 % CI, 1.18–2.42); *P* = 0.004) problem and possibly heart problem (RRR, 1.42 (95 % CI, 0.99–2.04); *P* = 0.056) were more dissatisfied with the community centres and facilities, compared with people without such health conditions (data now shown). People with vision (RRR, 1.36 (95 % CI, 1.02–1.80); *P* = 0.034) and neck (RRR, 1.36 (95 % CI, 1.11–1.66); *P* = 0.003) problem, other progressive illness (RRR, 1.43 (95 % CI, 1.03–2.00); *P* = 0.034), other disability (RRR, 1.36 (95 % CI, 1.01–1.81); *P* = 0.041) and possibly heart problem (RRR, 1.25 (95 % CI, 0.99–1.59); *P* = 0.059) were also more dissatisfied with the police service. Furthermore, people with vision (RRR, 1.43 (95 % CI, 1.10–1.85); *P* = 0.007) problem were dissatisfied with the local fire service.

## Discussion

### Waste management

Health hazards from waste management have been studied among waste management workers (Sigsgaard 1999). During sorting and recycling, there could be bioaerosol exposure (e.g. airborne bacteria, endotoxin etc.) revealed (Poulsen et al. 1995). The typical health risks are gastrointestinal symptoms, respiratory problems and irritation of the eyes and skin. In the present study, dissatisfaction among people with arthritis, chest or hearing problems was also observed. This might be as a result of local improper waste management leading to the impact on these people with the exciting health conditions or they have found it difficult/challenging for them to do refuse collection in the neighbourhood. Unfortunately, it is not possible to find out the real cause from the current limited dataset.

### Public transportation

Although the relationship of traffic (congestion) and mental health could have been less studied, compared with other neighbourhood risks such as air quality, crime, noise etc., a few community studies have observed that transport team members had higher incident mental health episodes while there was observed an association between high vehicle traffic density in residential area and reduced quality of life and mental health in women across several countries as well (Tvaryanas and Maupin 2014; Gundersen et al. 2013; Yamazaki et al. 2005). Primary school children could have suffered from transportation noise resulting in neuro-behavioural conditions (van Kempen et al. 2010). People with dyslexia could have been further impacted by the lack of clear aid in the public space leading to long time frustration in streets (Bentzen et al. 2007; Brachacki et al. 1995) or the loss of driving ability to adapt the rapid changing environments on roads (Sigmundsson 2005; Groeger and Maguire 1996). Following these observations and the results from the present study, a universal public transportation development plan to include the needs of people with dyslexia could be suggested.

### Street cleaning

It has been observed the links between air quality and health conditions such as heart disease, asthma and cancer (Ernst et al. 2003; Evans Kantrowitz 2002), in particular in populations with specific occupations (Biggi et al. 2008). In addition to regulating chemical emission from industry or buildings, a recent trial on intense street cleaning was found to be effective to lessen pollutants in public space and consequently health risk effects (Amato et al. 2010). Current urban design could have still ignored the complete consideration of well-functioning neighbourhoods (Jackson 2003). Such investment should therefore be put into environmental and social policies as to delay or prevent health problems that might deteriorate human capital in the long run.

### Strengths and limitations

The present study has a number of strengths. Firstly, it was conducted in a representative study sample (country-wide and population-based) and in recent years. Secondly, it was also the first time to analyse how people with long-term standing illness and disability could perceive their living neighbourhoods in large study sample in Scotland. However, there are also a few limitations worthy of being noted. First, the list of items in assessing satisfaction toward neighbourhood facilities was not standardised. Future studies including epidemiologists, architects and civil engineers working together from developing a complete questionnaire to managing built environment toward the universal design would be suggested. Second, although there were some significant associations observed, the statistical modelling could still have been suffering from small number of cases in some sub-scales. Third, the causality cannot be established due to the cross-sectional study design in nature. Taken together, future research retaining the strengths and overcoming these limitations mentioned above with a long-term monitoring would be suggested.

### Directions for future research, practice and policy

In sum, people with heart, chest, skin, digestive, musculoskeletal, vision, learning, speech and mental disorders and dyslexia were more dissatisfied with their current neighbourhood environments. For future research, studies moving from etiological factors to problematic neighbourhood management and restoration in a well-established surveillance for both urban and town reviving would be recommended in order to ensure the neighbourhood equality for all residents. For clinical practice, upgrading neighbourhood planning to tackle social environment injustice would be suggested in order to have a balanced focus on both places and people (Shiue 2016). For policy making, regular monitoring on the neighbourhood condition for proper maintenance and preservation might be necessary in order to ensure that the health and well-being of all residents could be maintained and optimised and no one would be left behind to amount health and social care services use exceedingly due to the vicious circle.

## References

[CR1] Amato F, Nava S, Lucarelli F, Querol X, Alastuey A, Baldadano JM, Pandolfi MA (2010). Comprehensive assessment of PM emissions from paved roads: real-world emission factors and intense street cleaning trials. Sci Total Environ.

[CR2] Bentzen BL, Crandall WF, Myers L (1671). Wayfinding system for transportation services: remote infrared audible signage for transit stations, surface transit, and intersections. J Transportation Res Board.

[CR3] Biggi N, Consonni D, Galluzzo V, Sogliani M, Costa G (2008). Metabolic syndrome in permanent night workers. Chronobiol Int.

[CR4] Bowling A, Farquhar M, Grundy E, Formby J (1993). Changes in life satisfaction over a two and a half year period among very elderly people living in London. Soc Sci Med.

[CR5] Brachacki GW, Nicolson RI, Fawcett AJ (1995) Impaired recognition of traffic signs in adults with dyslexia. J Learn Disabil 28:297–301 10.1177/002221949502800505 7775849

[CR6] Chapman NJ, Beaudet M (1983). Environmental predictors of well-being for at-risk older adults in a mid-sized city. J Gerontol.

[CR7] Ernst M, Corless J, Greene-Roesel R. Clearing the air: public health threats from cars and heavy duty vehicles—why we need to protect federal clean air laws. Surface Transportation Policy Project. 2003.

[CR8] Evans GW, Kantrowitz E (2002). Socioeconomic status and health: the potential role of environmental risk exposure. Annu Rev Public Health.

[CR9] Groeger JA, Maguire RL. Dyslexia and driving: controlled processing of control skills? In: Behavioural Research in Road Safety VI. Crowthorne, UK: Transport Research Laboratory, 109–116.

[CR10] Gundersen H, Magerøy N, Moen BE, Bråtveit M (2013). Traffic density in area of residence is associated with health-related quality of life in women, the community-based Hordaland health study. Arch Environ Occup Health.

[CR11] Jackson LE (2003). The relationship of urban design to human health and condition. Landsc Urban Plan.

[CR12] Poulsen OM1, Breum NO, Ebbehøj N, Hansen AM, Ivens UI, van Lelieveld D, Malmros P, Matthiasen L, Nielsen BH, Nielsen EM, Schibyea B, Skova T, Stenbaeka EI, Wilkinsa KC (1995). Sorting and recycling of domestic waste. Review of occupational health problems and their possible causes. Sci Total Environ.

[CR13] Reitzel LR, Nguyen N, Zafereo ME, Li G, Wei Q, Sturgis EM (2012). Neighborhood deprivation and clinical outcomes among head and neck cancer patients. Health Place.

[CR14] Shiue I (2014). Neighborhood epidemiological monitoring and adult mental health: European quality of life survey, 2007–2012. Environ Sci Pollut Res Int.

[CR15] Shiue I (2014). Prevalence and psychiatric correlates of neighbourhood satisfaction and its impact on adolescent behaviours: UK understanding society cohort, 2011–2012. Environ Res.

[CR16] Shiue I (2016). Future urban design strategies for health and wellbeing: proposal of DIDID action plan and design mapping. Journal of Engineering, Design and Technology.

[CR17] Sigmundsson H (2005). Do visual processing deficits cause problem on response time task for dyslexics?. Brain Cogn.

[CR18] Sigsgaard T (1999). Health hazards to waste management workers in Denmark. Schriftenr Ver Wasser Boden Lufthyg.

[CR19] Tvaryanas AP, Maupin GM (2014). Risk of incident mental health conditions among critical care air transport team members. Aviat Space Environ Med.

[CR20] van Kempen E, van Kamp I, Lebret E, Lammers J, Emmen H, Stansfeld S (2010) Neurobehavioral effects of transportation noise in primary schoolchildren: a cross-sectional study. Environ Health:9:25 10.1186/1476-069X-9-25 PMC2898757 20515466

[CR21] Warr D, Feldman P, Tacticos T, Kelaher M (2009). Sources of stress in impoverished neighbourhoods: insights into links between neighbourhood environments and health. Aust N Z J Public Health.

[CR22] Yamazaki S, Sokejima S, Nitta H, Nakayama T, Fukuhara S (2005). Living close to automobile traffic and quality of life in Japan: a population-based survey. Int J Environ Health Res.

